# *N*-[2-(Tri­fluoro­meth­yl)phen­yl]maleamic acid: crystal structure and Hirshfeld surface analysis

**DOI:** 10.1107/S2056989019006509

**Published:** 2019-05-10

**Authors:** P. A. Suchetan, Shet M. Prakash, N. K. Lokanath, S. Naveen, Ismail Warad

**Affiliations:** a Dept. of Chemistry, University College of Science, Tumkur University, Tumkur, 572 103, India; b Department of Studies in Physics, University of Mysore, Manasagangotri, Mysuru 570 006, India; c Department of Basic Sciences, School of Engineering and Technology, Jain, University, Bangalore 562 112, India; d Department of Chemistry, Science College, An-Najah National University, PO Box 7, Nablus, Palestinian Territories

**Keywords:** crystal structure, hydrogen bonds, maleamic acids, Hirshfeld surface

## Abstract

The title mol­ecule, C_11_H_8_F_3_NO_3_, adopts a *cis* configuration across the –C=C– double bond in the side chain and the dihedral angle between the phenyl ring and side chain is 47.35 (1)°. The –COOH group adopts a *syn* conformation (O=C—O—H = 0°), unlike the *anti* conformation observed in related maleamic acids. In the crystal, inversion dimers linked by pairs of O—H⋯O hydrogen bonds are connected *via* N—H⋯O hydrogen bonds and C—H⋯O inter­actions into (100) sheets, which are cross-linked by another C—H⋯O inter­action to result in a three-dimensional network. The Hirshfeld surface fingerprint plots show that the highest contribution to surface contacts arises from O⋯H/H⋯O contacts (26.5%) followed by H⋯F/F⋯H (23.4%) and H⋯H (17.3%).

## Abstract 2

The –COOH group of the title compound adopts a *syn* conformation (O= C—O—H = 0°) unlike the *anti* conformation observed in related maleamic acids. This is correlated with the formation of carb­oxy­lic acid inversion dimers linked by pairwise O—H⋯O hydrogen bonds in the crystal of the title compound rather than an intra­molecular O—H⋯O hydrogen bond.

## Chemical context   

The development of pH-induced charge-conversion drug-delivery systems can help to overcome the intrinsic pH difference between tumor tissues (pH 6.5–6.8) and normal tissues or the blood stream (pH 7.2–7.4) (Ge *et al.*, 2013 ▸). Reactions of 2,3-di­methyl­maleic anhydride (DMMA) and amino groups on the particle surface have been used to shield the positive charge of nanoparticles (Du *et al.*, 2010 ▸). The generated amide bond is cleavable under mildly acidic conditions but is stable at neutral or basic pH, whereas the DMMA-decorated nanoparticles are inert under physiological conditions. After accumulating into the acidic tumor tissue through the enhanced permeation and retention (EPR) effect, the amide bond slowly cleaves and thus exposes the positive charge, which eventually promotes cell inter­nalization. Therefore, maleamic acids and their derivatives, by virtue of their unique weak acid sensitivity and charge conversion have been widely used as smart carriers to deliver nucleic acids (Meyer *et al.*, 2009 ▸), proteins (Zhang *et al.*, 2015 ▸; Lee *et al.*, 2007 ▸) and drugs (Du *et al.*, 2011 ▸; Chen *et al.*, 2015 ▸; Han *et al.*, 2015 ▸). Simple methods to control the ratio of two positional isomers of mono-substituted maleamic acids and a highly efficient way to synthesize di-substituted maleamic acids have been reported (Su *et al.*, 2017 ▸). The hydrolysis profiles of mono- or di-substituted maleamic acids were studied by the same authors to elucidate their hydrolysis selectivity towards various physiologically available pH values (Su *et al.*, 2017 ▸). As part our studies in this area, the synthesis and crystal structure of N-[2-(tri­fluoro­meth­yl)phen­yl]maleamic acid, (I), is described and is further analysed using Hirshfeld surfaces and fingerprint plots and compared to related structures.
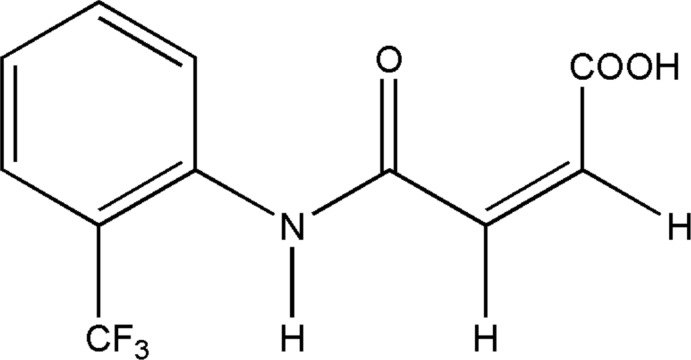


## Structural commentary   

**Figure 1:**
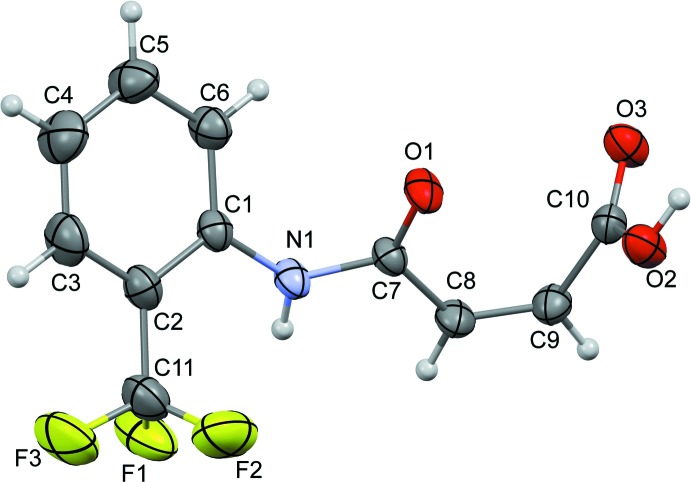
A view of the mol­ecular structure of (I), with displacement ellipsoids drawn at the 50% probability level.

The mol­ecule of (I) adopts a *cis* configuration across the –C=C– double bond in the side chain (Fig. 1 ▸), similar to that observed in *N*-(phen­yl)maleamic acid (Lo *et al.*, 2009 ▸) and other related *o*-substituted maleamic acids, *viz. N*-(2-methyl­phen­yl)maleamic acid (Gowda *et al.*, 2010 ▸) and *N*-(2-amino­phen­yl)maleamic acid (Santos-Sánchez *et al.*, 2007 ▸). In (I), the dihedral angle between the planes of the phenyl ring C1–C6 and the side chain C1—N1(O1)—C7—C8—C9 is 47.35 (1)° compared to the reported values of 12.7 (1)° in *N*-(2-methyl­phen­yl)maleamic acid (Gowda *et al.*, 2010 ▸) and 43.08 (10)° in *N*-(2-amino­phen­yl)maleamic acid (Santos-Sánchez *et al.*, 2007 ▸). Compound (I) differs from related structures in the conformation of its carb­oxy­lic acid group. In (I), the –COOH group adopts *syn* conformation (*i.e*. the O3=C10—O2—H2*O* torsion angle = 0°) whereas an *anti* conformation is noted in related structures (the equivalent torsion angle is close to 180°). This disparity is a result of O—H_c_⋯O=C_a_ (c = carb­oxy­lic acid, a = amide) intra­molecular hydrogen bonds present in related structures and not observed in (I).

## Supra­molecular features   

**Figure 2:**
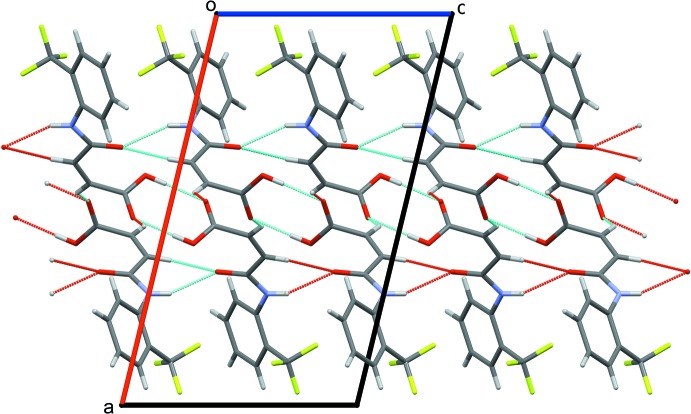
A view down [010] of the crystal packing in (I) showing the sheets of mol­ecules linked by O—H⋯O and N—H⋯O hydrogen bonds and C—H⋯O inter­actions (thin blue lines).

In the crystal of (I), the mol­ecules are connected *via* pairwise O2—H2*O*⋯O3 hydrogen bonds (Fig. 2 ▸, Table 1 ▸) forming 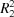
(8) inversion dimers and N1—H1*N*⋯O1 hydrogen bonds forming *C*(4) chains (Fig. 2 ▸, Table 1 ▸), resulting in sheets lying in the (100) plane (Fig. 2 ▸). The N1—H1*N*⋯O1 hydrogen bond is reinforced by a C8—H8⋯O1 inter­action forming another *C*(4) chain in its own right (Fig. 2 ▸, Table 1 ▸). In addition, C9—H9⋯O3 inter­actions (Table 1 ▸) forming *C*(4) chains runs down the *b*-axis direction, thereby cross-linking the sheets into a three-dimensional network.

## Hirshfeld surface analysis   

**Figure 3:**
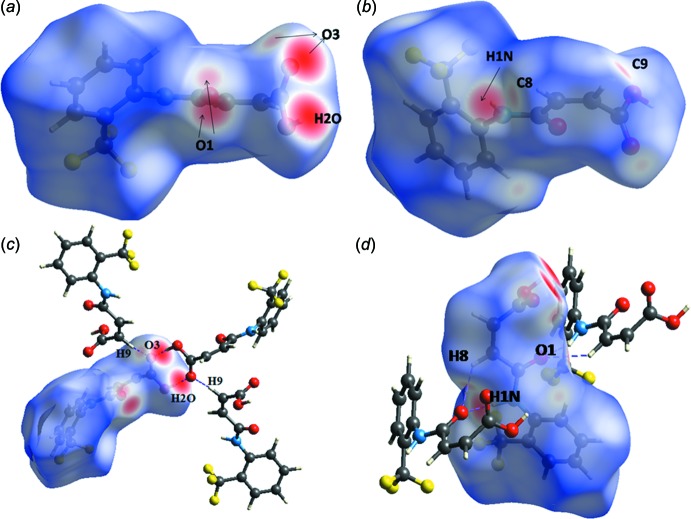
The Hirshfeld surface mapped with *d*_norm_ for the mol­ecule in (I) over the range −0.753 to 1.252 a.u., shown inter­acting with near-neighbour mol­ecules connected through hydrogen bonds (dashed lines).

**Figure 4:**
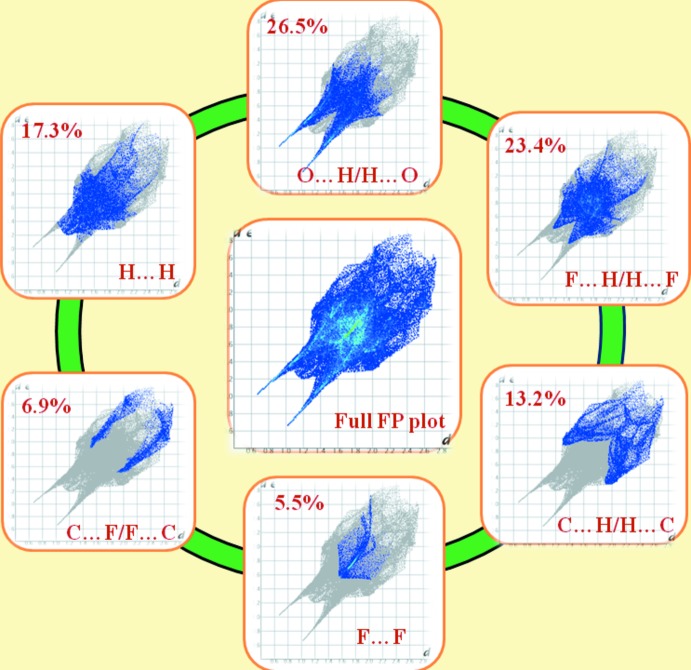
The full two-dimensional fingerprint plot and those delineated into O⋯H/H⋯O, F⋯H/H⋯F, H⋯H, C⋯H/H⋯C, C⋯F/F⋯C and F⋯F contacts in (I).

In the Hirshfeld surface analysis, *d*_norm_ surfaces and two-dimensional fingerprint plots (FP) were generated to further investigate the inter­molecular inter­actions in (I) and to provide qu­anti­tative data for the relative contributions to the surfaces (Turner *et al.*, 2017 ▸). The appearance of both dark- and faint-red spots near O1 and O3 support the involvement of each of these atoms in architectures involving the acceptance of a strong hydrogen bond and a weak inter­molecular inter­action (Fig. 3 ▸). Similarly, dark-red spots near the H1*N* and H2*O* hydrogen atoms are due to their involvement as donors in stronger hydrogen bonds, while faint spots near H8 and H9 atoms are due to the weak C—H⋯O inter­actions involving these atoms (Fig. 3 ▸). Analysis of the fingerprint plots (Fig. 4 ▸) showed that the major contributions to the overall Hirshfeld surfaces of (I) are from O⋯H/H⋯O (26.5%; *d*_i_ + *d*_e_ ∼1.8 Å), F⋯H/H⋯F (23.4%; *d*_i_ + *d*_e_ ∼2.6 Å), H⋯H (17.3%; *d*_i_ + *d*_e_ ∼2.4 Å), C⋯H/H⋯C (13.2%; *d*_i_ + *d*_e_ ∼3.2 Å), C⋯F/F⋯C (6.9%; *d*_i_ + *d*_e_ ∼3.4 Å) and F⋯F (5.5%; *d*_i_ + *d*_e_ ∼3.2 Å) inter­actions, with other contacts contributing the remaining 10.2%.

## Database survey   

Nineteen *N*-(ar­yl)-maleamic acids have been reported to date with varied substituents (mono-, di- and tris­ubstituted derivatives at different positions) on the phenyl ring. Three of these, namely *N*-(phen­yl)maleamic acid (CCDC refcode: LOSJUZ) (Lo *et al.*, 2009 ▸) and two *o*-substituted compounds, *viz. N*-(2-methyl­phen­yl)maleamic acid (QUYJUQ) (Gowda *et al.*, 2010 ▸) and *N*-(2-amino­phen­yl)maleamic acid (PILVAI) (Santos-Sánchez *et al.*, 2007 ▸) are closely related to (I), and are therefore of most relevance to the present work. The other 16 structures are either di/tri-substituted compounds or monosubstituted ones at the *meta*/*para* positions. The nature and type of inter­molecular inter­actions, and thereby the resulting architecture in (I) is different from those observed in the three structures, which each feature an *anti* O=C—O—H conform­ation and an intra­molecular O—H⋯O hydrogen bond, as noted above. In LOSJUZ, adjacent mol­ecules are linked by N—H⋯O hydrogen bonds into a flat ribbon, while in QUYJUQ, N—H⋯O hydrogen bonds link the mol­ecules into zigzag chains propagating parallel to [001] and these chains are further linked into sheets by weak π–π inter­actions. In the crystal structure of PILVAI, symmetry-related mol­ecules are linked by N—H⋯N hydrogen bonds, forming centrosymmetric amine–amide dimers. The dimers are linked by N—H⋯O and C—H⋯O hydrogen bonds and weak N—H⋯π and π–π inter­actions into a three-dimensional network.

## Synthesis and crystallization   

The title compound was synthesized by following the same procedure that was employed for synthesizing *N*-(2-methyl­phen­yl)maleamic acid (Gowda *et al.*, 2010 ▸). Colourless prisms of (I) were recrystallized from ethanol solution.

## Refinement   

Crystal data, data collection and structure refinement details are summarized in Table 2 ▸. The carbon-bound H atoms were placed in calculated positions (C—H = 0.93 Å) and were included in the refinement in the riding-model approximation, with *U*_iso_(H) set to 1.2*U*_eq_(C). The oxygen- and nitro­gen-bound H atoms were located from difference-Fourier maps and freely refined.

## Supplementary Material

Crystal structure: contains datablock(s) I. DOI: 10.1107/S2056989019006509/hb7816sup1.cif

Structure factors: contains datablock(s) I. DOI: 10.1107/S2056989019006509/hb7816Isup2.hkl

Supporting information file. DOI: 10.1107/S2056989019006509/hb7816Isup3.cml

CCDC reference: 1914411

Additional supporting information:  crystallographic information; 3D view; checkCIF report

## Figures and Tables

**Table 1:** Hydrogen-bond geometry (Å, °)

*D*—H⋯*A*	*D*—H	H⋯*A*	*D*⋯*A*	*D*—H⋯*A*
N1—H1*N*⋯O1^i^	0.86 (2)	2.15 (2)	2.937 (3)	153 (3)
O2—H2*O*⋯O3^ii^	0.84 (2)	1.84 (2)	2.679 (3)	179 (7)
C8—H8⋯O1^i^	0.93	2.48	3.213 (3)	136
C9—H9⋯O3^iii^	0.93	2.49	3.190 (4)	133

Symmetry codes: (i) 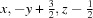; (ii) 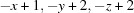; (iii) 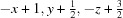.

**Table 2:** Experimental details

Crystal data	
Chemical formula	C_11_H_8_F_3_NO_3_
*M*_r_	259.18
Crystal system, space group	Monoclinic, *P*2_1_/*c*
Temperature (K)	293
*a*, *b*, *c* (Å)	16.307 (4), 7.6438 (16), 9.532 (2)
β (°)	103.669 (8)
*V* (Å^3^)	1154.5 (4)
*Z*	4
Radiation type	Mo *K*α
μ (mm^−1^)	0.14
Crystal size (mm)	0.22 × 0.19 × 0.17

Data collection	
Diffractometer	Bruker APEXII CCD
Absorption correction	Multi-scan (*SADABS*; Bruker, 2009 ▸)
*T*_min_, *T*_max_	0.970, 0.976
No. of measured, independent and observed [*I* > 2σ(*I*)] reflections	4213, 2598, 1690
*R*_int_	0.061
(sin θ/λ)_max_ (Å^−1^)	0.650

Refinement	
*R*[*F* ^2^ > 2σ(*F* ^2^)], *wR*(*F* ^2^), *S*	0.065, 0.186, 1.03
No. of reflections	2598
No. of parameters	171
No. of restraints	2
H-atom treatment	H atoms treated by a mixture of independent and constrained refinement
Δρ_max_, Δρ_min_ (e Å^−3^)	0.34, −0.26

Computer programs: *APEX2* and *SAINT-Plus* (Bruker, 2009 ▸), *SHELXT2016* (Sheldrick, 2015*a* ▸), *SHELXL2016* (Sheldrick, 2015*b* ▸) and *Mercury* (Macrae *et al.*, 2008 ▸).

## supplementary crystallographic information

### Crystal data

**Table:**

C_11_H_8_F_3_NO_3_	Prism
*M**_r_* = 259.18	*D*_x_ = 1.491 Mg m^−^^3^
Monoclinic, *P*2_1_/*c*	Melting point: 440 K
Hall symbol: -P 2ybc	Mo *K*α radiation, λ = 0.71073 Å
*a* = 16.307 (4) Å	Cell parameters from 143 reflections
*b* = 7.6438 (16) Å	θ = 3.5–27.5°
*c* = 9.532 (2) Å	µ = 0.14 mm^−^^1^
β = 103.669 (8)°	*T* = 293 K
*V* = 1154.5 (4) Å^3^	Prism, colourless
*Z* = 4	0.22 × 0.19 × 0.17 mm
*F*(000) = 528	

### Data collection

**Table:**

Bruker APEXII CCD diffractometer	2598 independent reflections
Radiation source: fine-focus sealed tube	1690 reflections with *I* > 2σ(*I*)
Graphite monochromator	*R*_int_ = 0.061
ω scans	θ_max_ = 27.5°, θ_min_ = 3.5°
Absorption correction: multi-scan (SADABS; Bruker, 2009)	*h* = −21→12
*T*_min_ = 0.970, *T*_max_ = 0.976	*k* = −9→9
4213 measured reflections	*l* = −12→11

### Refinement

**Table:**

Refinement on *F*^2^	Primary atom site location: dual
Least-squares matrix: full	Hydrogen site location: mixed
*R*[*F*^2^ > 2σ(*F*^2^)] = 0.065	H atoms treated by a mixture of independent and constrained refinement
*wR*(*F*^2^) = 0.186	*w* = 1/[σ^2^(*F*_o_^2^) + (0.0701*P*)^2^ + 0.4963*P*] where *P* = (*F*_o_^2^ + 2*F*_c_^2^)/3
*S* = 1.02	(Δ/σ)_max_ < 0.001
2598 reflections	Δρ_max_ = 0.34 e Å^−^^3^
171 parameters	Δρ_min_ = −0.26 e Å^−^^3^
2 restraints	

### Special details

**Table:**

Geometry. All esds (except the esd in the dihedral angle between two l.s. planes) are estimated using the full covariance matrix. The cell esds are taken into account individually in the estimation of esds in distances, angles and torsion angles; correlations between esds in cell parameters are only used when they are defined by crystal symmetry. An approximate (isotropic) treatment of cell esds is used for estimating esds involving l.s. planes.

### Fractional atomic coordinates and isotropic or equivalent isotropic displacement parameters (Å^2^)

**Table:**

	*x*	*y*	*z*	*U*_iso_*/*U*_eq_	
O1	0.34076 (13)	0.7544 (3)	0.73122 (17)	0.0570 (5)	
O2	0.41280 (13)	1.1030 (3)	0.8624 (2)	0.0604 (6)	
O3	0.52092 (13)	0.9298 (3)	0.8536 (2)	0.0609 (5)	
N1	0.28493 (13)	0.6788 (3)	0.4975 (2)	0.0475 (5)	
F2	0.15299 (15)	0.6837 (4)	0.2366 (2)	0.1062 (8)	
F3	0.03339 (14)	0.6184 (4)	0.2675 (3)	0.1295 (11)	
C1	0.23369 (16)	0.5362 (3)	0.5229 (2)	0.0457 (6)	
F1	0.1069 (2)	0.8137 (3)	0.3972 (3)	0.1357 (12)	
C7	0.33398 (15)	0.7774 (3)	0.6013 (2)	0.0425 (6)	
C10	0.45863 (17)	1.0115 (3)	0.7934 (3)	0.0465 (6)	
C9	0.43322 (18)	1.0235 (4)	0.6336 (3)	0.0523 (7)	
H9	0.457140	1.113209	0.590732	0.063*	
C8	0.38002 (17)	0.9193 (3)	0.5470 (3)	0.0502 (6)	
H8	0.371000	0.935384	0.447771	0.060*	
C6	0.26681 (18)	0.4086 (4)	0.6223 (3)	0.0580 (7)	
H6	0.322621	0.416324	0.674408	0.070*	
C2	0.15006 (17)	0.5244 (4)	0.4447 (3)	0.0546 (7)	
C3	0.1014 (2)	0.3826 (5)	0.4683 (4)	0.0756 (9)	
H3	0.045544	0.373178	0.416789	0.091*	
C5	0.2173 (2)	0.2686 (5)	0.6450 (4)	0.0761 (10)	
H5	0.239695	0.183177	0.712740	0.091*	
C11	0.1111 (2)	0.6602 (5)	0.3390 (4)	0.0750 (9)	
C4	0.1357 (2)	0.2570 (5)	0.5674 (4)	0.0861 (11)	
H4	0.102772	0.162430	0.581975	0.103*	
H1N	0.285 (2)	0.717 (4)	0.413 (2)	0.072 (10)*	
H2O	0.433 (3)	1.094 (6)	0.952 (2)	0.104 (14)*	

### Atomic displacement parameters (Å^2^)

**Table:**

	*U*^11^	*U*^22^	*U*^33^	*U*^12^	*U*^13^	*U*^23^
O1	0.0721 (13)	0.0702 (12)	0.0297 (8)	−0.0182 (10)	0.0138 (8)	−0.0043 (8)
O2	0.0620 (12)	0.0664 (13)	0.0500 (11)	0.0158 (10)	0.0073 (9)	−0.0087 (10)
O3	0.0565 (12)	0.0719 (13)	0.0507 (10)	0.0130 (10)	0.0051 (9)	−0.0098 (9)
N1	0.0513 (12)	0.0585 (13)	0.0319 (10)	−0.0109 (10)	0.0085 (8)	−0.0007 (10)
F2	0.0939 (16)	0.152 (2)	0.0649 (12)	−0.0081 (15)	0.0031 (11)	0.0347 (13)
F3	0.0652 (14)	0.161 (2)	0.136 (2)	−0.0054 (16)	−0.0303 (13)	0.0407 (19)
C1	0.0465 (14)	0.0539 (14)	0.0380 (11)	−0.0066 (12)	0.0127 (10)	−0.0059 (11)
F1	0.188 (3)	0.0863 (17)	0.1125 (19)	0.0550 (19)	−0.0055 (18)	0.0035 (15)
C7	0.0436 (13)	0.0517 (13)	0.0319 (11)	−0.0027 (11)	0.0084 (9)	−0.0030 (10)
C10	0.0488 (14)	0.0410 (12)	0.0483 (13)	−0.0048 (11)	0.0089 (11)	−0.0058 (11)
C9	0.0653 (17)	0.0480 (15)	0.0452 (13)	−0.0079 (13)	0.0163 (12)	0.0014 (11)
C8	0.0610 (16)	0.0553 (15)	0.0339 (11)	−0.0063 (13)	0.0104 (11)	0.0031 (11)
C6	0.0467 (15)	0.0666 (18)	0.0597 (16)	−0.0050 (14)	0.0104 (12)	0.0043 (14)
C2	0.0421 (14)	0.0652 (17)	0.0542 (15)	−0.0019 (13)	0.0071 (11)	−0.0063 (14)
C3	0.0479 (16)	0.088 (2)	0.086 (2)	−0.0175 (17)	0.0066 (15)	−0.003 (2)
C5	0.066 (2)	0.071 (2)	0.091 (2)	−0.0053 (17)	0.0175 (17)	0.0224 (19)
C11	0.0561 (19)	0.093 (3)	0.0674 (19)	−0.0018 (19)	−0.0028 (15)	0.005 (2)
C4	0.067 (2)	0.079 (2)	0.112 (3)	−0.0220 (19)	0.020 (2)	0.014 (2)

### Geometric parameters (Å, º)

**Table:**

O1—C7	1.230 (3)	C10—C9	1.483 (4)
O2—C10	1.309 (3)	C9—C8	1.316 (4)
O2—H2O	0.845 (18)	C9—H9	0.9300
O3—C10	1.215 (3)	C8—H8	0.9300
N1—C7	1.346 (3)	C6—C5	1.388 (4)
N1—C1	1.428 (3)	C6—H6	0.9300
N1—H1N	0.861 (18)	C2—C3	1.393 (4)
F2—C11	1.328 (4)	C2—C11	1.481 (4)
F3—C11	1.329 (4)	C3—C4	1.369 (5)
C1—C6	1.378 (4)	C3—H3	0.9300
C1—C2	1.394 (4)	C5—C4	1.365 (5)
F1—C11	1.307 (4)	C5—H5	0.9300
C7—C8	1.481 (3)	C4—H4	0.9300
			
C10—O2—H2O	110 (3)	C1—C6—H6	119.8
C7—N1—C1	124.9 (2)	C5—C6—H6	119.8
C7—N1—H1N	112 (2)	C1—C2—C3	119.1 (3)
C1—N1—H1N	123 (2)	C1—C2—C11	121.8 (3)
C6—C1—C2	119.8 (2)	C3—C2—C11	119.1 (3)
C6—C1—N1	120.4 (2)	C4—C3—C2	120.1 (3)
C2—C1—N1	119.8 (2)	C4—C3—H3	119.9
O1—C7—N1	123.8 (2)	C2—C3—H3	119.9
O1—C7—C8	121.6 (2)	C4—C5—C6	119.6 (3)
N1—C7—C8	114.6 (2)	C4—C5—H5	120.2
O3—C10—O2	123.4 (2)	C6—C5—H5	120.2
O3—C10—C9	121.1 (2)	F1—C11—F3	107.1 (3)
O2—C10—C9	115.4 (2)	F1—C11—F2	106.2 (4)
C8—C9—C10	126.1 (2)	F3—C11—F2	104.4 (3)
C8—C9—H9	116.9	F1—C11—C2	113.4 (3)
C10—C9—H9	116.9	F3—C11—C2	112.6 (3)
C9—C8—C7	122.5 (2)	F2—C11—C2	112.5 (3)
C9—C8—H8	118.7	C3—C4—C5	121.0 (3)
C7—C8—H8	118.7	C3—C4—H4	119.5
C1—C6—C5	120.4 (3)	C5—C4—H4	119.5
			
C7—N1—C1—C6	−48.6 (4)	C6—C1—C2—C11	178.7 (3)
C7—N1—C1—C2	132.6 (3)	N1—C1—C2—C11	−2.5 (4)
C1—N1—C7—O1	0.7 (4)	C1—C2—C3—C4	0.0 (5)
C1—N1—C7—C8	−179.1 (2)	C11—C2—C3—C4	−178.9 (3)
O3—C10—C9—C8	92.9 (4)	C1—C6—C5—C4	0.6 (5)
O2—C10—C9—C8	−91.3 (4)	C1—C2—C11—F1	−63.2 (4)
C10—C9—C8—C7	3.4 (5)	C3—C2—C11—F1	115.7 (4)
O1—C7—C8—C9	3.0 (4)	C1—C2—C11—F3	175.0 (3)
N1—C7—C8—C9	−177.3 (3)	C3—C2—C11—F3	−6.1 (5)
C2—C1—C6—C5	−0.1 (4)	C1—C2—C11—F2	57.3 (4)
N1—C1—C6—C5	−178.9 (3)	C3—C2—C11—F2	−123.8 (3)
C6—C1—C2—C3	−0.2 (4)	C2—C3—C4—C5	0.4 (6)
N1—C1—C2—C3	178.6 (3)	C6—C5—C4—C3	−0.7 (6)

### Hydrogen-bond geometry (Å, º)

**Table:**

*D*—H···*A*	*D*—H	H···*A*	*D*···*A*	*D*—H···*A*
N1—H1*N*···O1^i^	0.86 (2)	2.15 (2)	2.937 (3)	153 (3)
O2—H2*O*···O3^ii^	0.84 (2)	1.84 (2)	2.679 (3)	179 (7)
C8—H8···O1^i^	0.93	2.48	3.213 (3)	136
C9—H9···O3^iii^	0.93	2.49	3.190 (4)	133

Symmetry codes: (i) *x*, −*y*+3/2, *z*−1/2; (ii) −*x*+1, −*y*+2, −*z*+2; (iii) −*x*+1, *y*+1/2, −*z*+3/2.
